# Intracellular Pharmacodynamic Modeling Is Predictive of the Clinical Activity of Fluoroquinolones against Tuberculosis

**DOI:** 10.1128/AAC.00989-19

**Published:** 2019-12-20

**Authors:** Samantha Donnellan, Ghaith Aljayyoussi, Emmanuel Moyo, Alison Ardrey, Carmen Martinez-Rodriguez, Stephen A. Ward, Giancarlo A. Biagini

**Affiliations:** a Centre for Drugs and Diagnostics, Department of Tropical Disease Biology, Liverpool School of Tropical Medicine, Pembroke Place, Liverpool, United Kingdom; b Institute of Infection and Global Health, University of Liverpool, Liverpool, United Kingdom

**Keywords:** PDi, infectious disease, pharmacodynamics, pharmacokinetics, preclinical drug studies, tuberculosis

## Abstract

Clinical studies of new antitubercular drugs are costly and time-consuming. Owing to the extensive tuberculosis (TB) treatment periods, the ability to identify drug candidates based on their predicted clinical efficacy is vital to accelerate the pipeline of new therapies. Recent failures of preclinical models in predicting the activity of fluoroquinolones underline the importance of developing new and more robust predictive tools that will optimize the design of future trials. Here, we used high-content imaging screening and pharmacodynamic intracellular (PDi) modeling to identify and prioritize fluoroquinolones for TB treatment. In a set of studies designed to validate this approach, we show moxifloxacin to be the most effective fluoroquinolone, and PDi modeling-based Monte Carlo simulations accurately predict negative culture conversion (sputum sterilization) rates compared to eight independent clinical trials. In addition, PDi-based simulations were used to predict the risk of relapse. Our analyses show that the duration of treatment following culture conversion can be used to predict the relapse rate. These data further support that PDi-based modeling offers a much-needed decision-making tool for the TB drug development pipeline.

## Abstract (precis)

Clinical studies of new antitubercular drugs are costly and time-consuming. Owing to the extensive tuberculosis (TB) treatment periods, the ability to identify drug candidates based on their predicted clinical efficacy is vital to accelerate the pipeline of new therapies. Recent failures of preclinical models in predicting the activity of fluoroquinolones underline the importance of developing new and more robust predictive tools that will optimize the design of future trials.

## INTRODUCTION

Tuberculosis (TB) caused by Mycobacterium tuberculosis is the leading cause of death from a single infectious agent. The so-called short course treatment of drug-susceptible TB (2-month intensive phase of rifampin [RIF], isoniazid [INH], pyrazinamide [PZA], and ethambutol [EMB], followed by 4 months of RIF and INH) (1) remains long, complex, and expensive, with a relatively high failure rate due to patient noncompliance and drug resistance. Fluoroquinolones were introduced into the regimen for multidrug-resistant TB (MDR-TB) after demonstration of their *in vitro* and *in vivo* antimycobacterial activity (2–6). They are now considered by the World Health Organization (WHO) to be a critical component in MDR-TB treatment. Fluoroquinolones are also administered when patients cannot tolerate the standard regimen (7). However, fluoroquinolones, especially third generation, are often discussed in terms of a generic drug class, and the specific choice of fluoroquinolone is generally not specified and is therefore often based on availability, cost, and national guidelines.

It was anticipated that by introducing fluoroquinolones into drug-susceptible regimens, the treatment period could be reduced by 2 months (from 6 to 4 months) (8). Clinical trials with moxifloxacin (MXF) produced mixed results, with some displaying superior activity that would indicate shorter treatment courses (9, 10) but others displaying little to no acceleration in achieving negative culture conversion (11, 12). However, a recent meta-analysis of all clinical data showed a significant improvement of culture conversion rates in total (13).

Pharmacokinetic/pharmacodynamic (PK/PD) models are useful in evaluating the length of treatment required by new regimens of anti-TB drugs. Recent clinical trials have highlighted the concerns of preclinical studies. Animal models show dynamics that differ from those observed in humans (14, 15), and predicted treatment improvements observed in mice with reformed regimens have often failed to reflect similar results in clinical settings. For example, murine studies with rifamycins (16–18) overpredicted the superior activity of higher doses of rifapentine in clinical studies. Similarly, animal studies were interpreted as showing a significant potential for the reduction in time of TB treatment with MXF (19), suggesting that treatment could be reduced by 1 month based on the *in vivo* results (20). However, phase 3 clinical trials (e.g., REMOX and RIFAQUIN) highlighted that despite the superiority of MXF, it was insufficient to display relapse-free cure rates observed in 6 months of conventional TB therapy (11, 21).

We have previously shown that PD data obtained from an *in vitro* intracellular (macrophage) *M. tuberculosis* high-content imaging-based platform, termed intracellular pharmacodynamics (PDi), can be a powerful tool for predicting the activity of first-line TB drugs in patients (22). Our platform is capable of defining the killing kinetics of first line anti-TB drugs against intracellular *M. tuberculosis*. Building from this previous work, and using a refined method that allows for extended monitoring of live drug-exposed intracellular *M. tuberculosis*, we profile fluoroquinolones here to assess their antitubercular efficacy. In addition, using these data we performed PDi-based PK/PD predictions of clinical outcome in terms of culture conversion rates and compare these values with clinical studies. The data are discussed in the context of the use of fluoroquinolones toward shortening treatment duration and the value of the PDi-based approach as a decision-making tool in the drug development of new treatment therapies.

## RESULTS

### Fluoroquinolones exhibit comparable rates of kill against intracellular *M. tuberculosis* but differ in potency.

**FIG 1:**
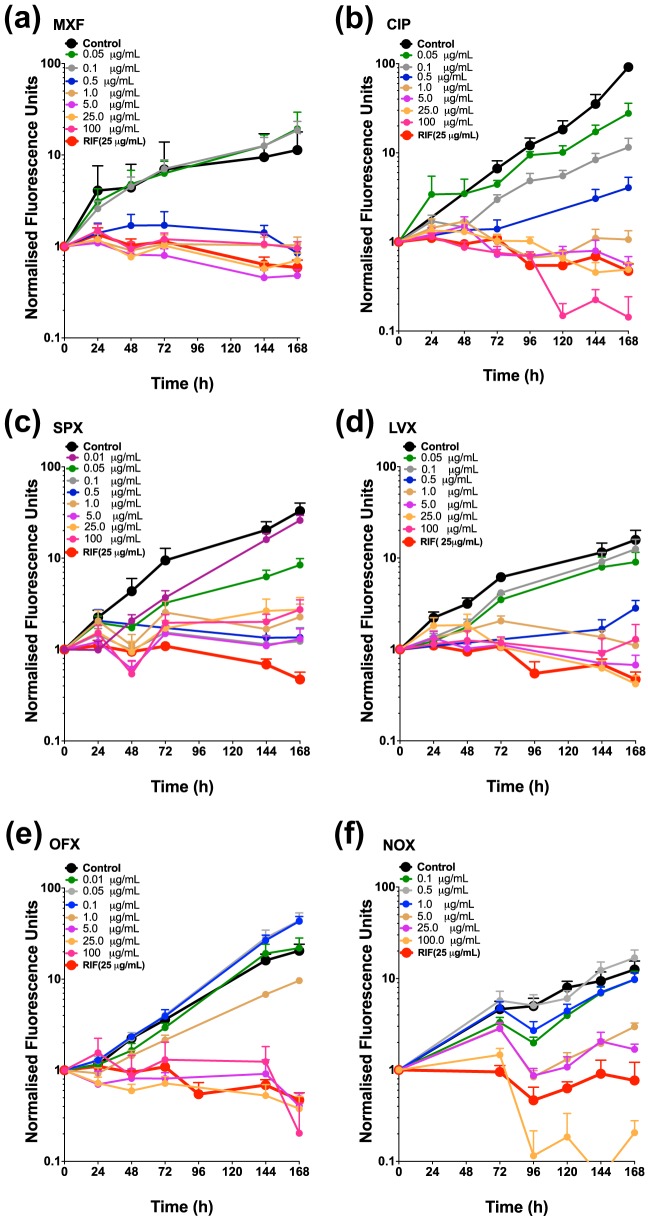
Intracellular (macrophage) *M. tuberculosis* time-dependent kill profiles of fluoroquinolones. Panels display time-kill profiles of MXF (a), CIP (b), SPX (c), LVX (d), OFX (e), and NOX (f). *M. tuberculosis* control (no drug) data are indicated in black, and RIF data at 25 mg/liter are indicated in red. The data are means ± the standard deviation (SD) derived from multiple independent experiments (*n* ≥ 3) performed at least in triplicate.

**FIG 2:**
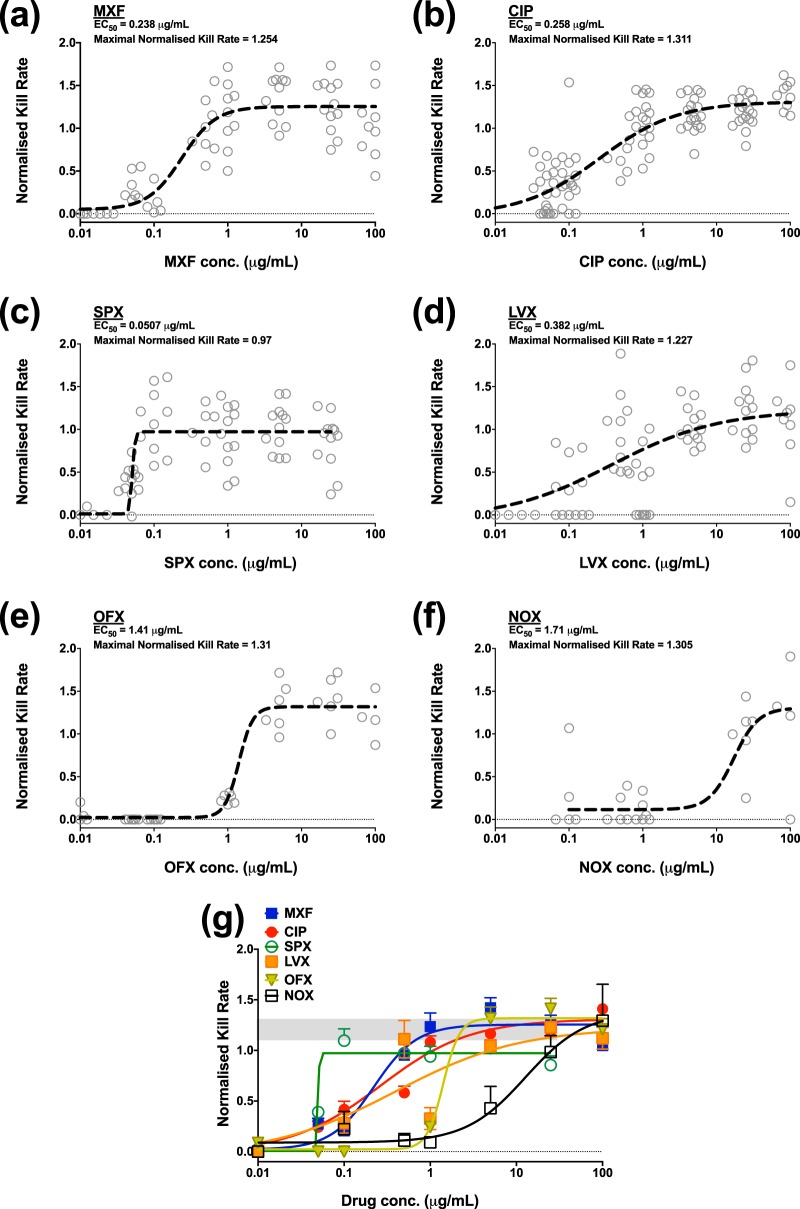
Concentration-intracellular *M. tuberculosis* kill rate relationship for selected fluoroquinolones. Open black circles represent individual kill rates at each concentration for drugs, and the dashed black lines display the three-parameter pharmacological fit for each drug. (a) MXF; (b) CIP; (c) SPX; (d) LVX; (e) OFX; (f) NOX. (g) Comparison of the profiles of all drugs. The gray area represents the kill rate of RIF at 25 mg/liter. *n* = 6 in three independent experimental replicates for each drug.

The efficacy of selected fluoroquinolones against intracellular *M. tuberculosis* was initially determined using the described fluorimetric-based assay (see methods). The intracellular anti-tubercular activity of MXF, levofloxacin (LVX), norfloxacin (NOX), ofloxacin (OFX), sparfloxacin (SPX), and ciprofloxacin (CIP) was assessed at a concentration ranges between 0.01 and 100 mg/liter. The kill rate elicited by each concentration was then calculated as previously described (22). Figure 1 displays the kill rate for each drug’s concentration range. A concentration-effect relationship using a three-parameter pharmacological model was then determined for each drug (Fig. 2). This model affords the calculation of pharmacological parameters, namely, the 50% effective concentration (EC_50_) and the maximal kill rates for each drug (Fig. 2a to f). Figure 2g compares the profiles of all drugs where the gray area represents the kill rate of RIF (a first-line antibiotic) at 25 mg/liter (this concentration was chosen since it is 1,000-fold the EC_50_ and is used to determine the *E*_max_ [see Materials and Methods]).

The fluoroquinolones tested displayed variability in potency for their respective intracellular anti-TB activities. SPX displayed the lowest EC_50_ (0.051 mg/liter), followed by MXF, CIP, and LVX (0.238, 0.259, and 0.382 mg/liter, respectively), while OFX and NOX displayed poor activity with EC_50_s of 1.414 to 1.705 mg/liter, as shown in Fig. 1 and 2 and in Table S1 in the supplemental material. Of note, although fluoroquinolones displayed variation in EC_50_s, the maximal kill rate for all fluoroquinolones was determined to be comparable (Fig. 2g), which is consistent with the drugs possessing the same mode of action.

Similar to our previous work with RIF, INH, and EMB (22), the MXF kill rate of *M. tuberculosis* grown in culture, termed extracellular, was significantly faster than the kill rate of MXF against intracellular (macrophage) *M. tuberculosis* (0.23 h^−1^ extracellular versus 0.055 h^−1^ intracellular [Fig. S1, and Fig. 1, respectively]).

### Determination of the *M. tuberculosis* growth/kill rate ratio for fluoroquinolones.

**FIG 3:**
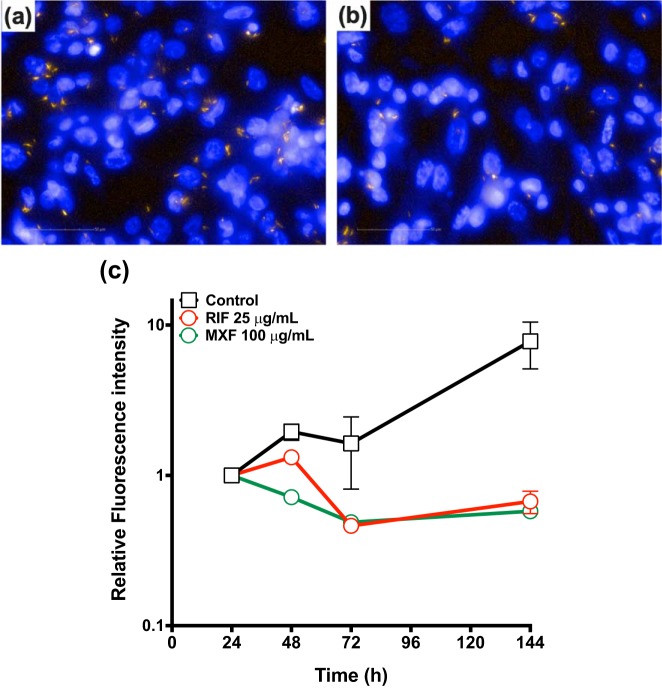
High-content fluorescent images as acquired from the Operetta (Perkin-Elmer). Fixed macrophages were infected with *M. tuberculosis* H37Rv expressing the far-red reporter mCherry after 72 h with no drug treatment (a) or with 100 mg/liter MXF (b). Orange, H37Rv-mCherry; blue, macrophage nuclei stained with Hoechst. Scale bar, 50 μm. (c) Activity of MXF against intracellular *M. tuberculosis* (data acquired from the Operetta via Harmony [Perkin-Elmer]). The red solid line displays the maximal kill rate obtained by RIF at 25 mg/liter.

The above-described live *M. tuberculosis* fluorimetry-based assay allowed us to rapidly determine the kill rate EC_50_ for the described fluoroquinolones, using a population-based fluorometric readout of intracellular *M. tuberculosis*. To define the maximal killing rate for the fluoroquinolone class at greater resolution and dynamic range, killing dynamics were measured using an Operetta-based high-content imaging screen, which can image individual bacilli residing inside macrophages (Fig. 3).

Figure 3 displays intracellular *M. tuberculosis* in the absence (Fig. 3a) or presence (Fig. 3b) of MXF (×63 magnification) and shows the maximal kill rate displayed by MXF (100 mg/liter) compared to RIF at a concentration of 25 mg/liter (Fig. 3c). The maximal kill rate of both drugs was equivalent and was determined to be approximately −1.8 × no-drug control growth rate. This concurs with the maximal kill rate/growth rate ratio previously reported for RIF (22). Our results demonstrate that despite RIF being the superior compound, by way of a lower EC_50_ value (0.019 mg/liter RIF versus 0.238 mg/liter MXF), the kill rate/growth rate ratio of MXF at a maximal concentration (100 mg/liter) was 1.82, similar to that of RIF at a maximal concentration (Fig. 3 and see Fig. S2 and S3 in the supplemental material).

### PDi modeling predicts culture conversion rates compared to clinical trial data.

MXF data from an Operetta-based high-content imaging screen was modeled and compared to clinical trial data reported in the literature. PDi modeling was performed for MXF using PD data obtained from the Operetta study. PK data and pulmonary exposure were calculated for MXF as reported when administered concomitantly with RIF since all simulations assumed concomitant administration of the two drugs (23, 24). Epithelial lining fluid (ELF) concentrations were used as a surrogate for pulmonary exposure and were obtained from the literature as estimated in healthy volunteers (25, 26). In the absence of such ratios in TB patients, we used healthy volunteer data as the closest possible estimate.

PDi modeling was used to generate COX regression curves. The curves indicate the percentage of patients achieving culture-negative conversion over time (culture conversion to negative is defined as <10 CFU/ml in sputum tests) (27).

**FIG 4:**
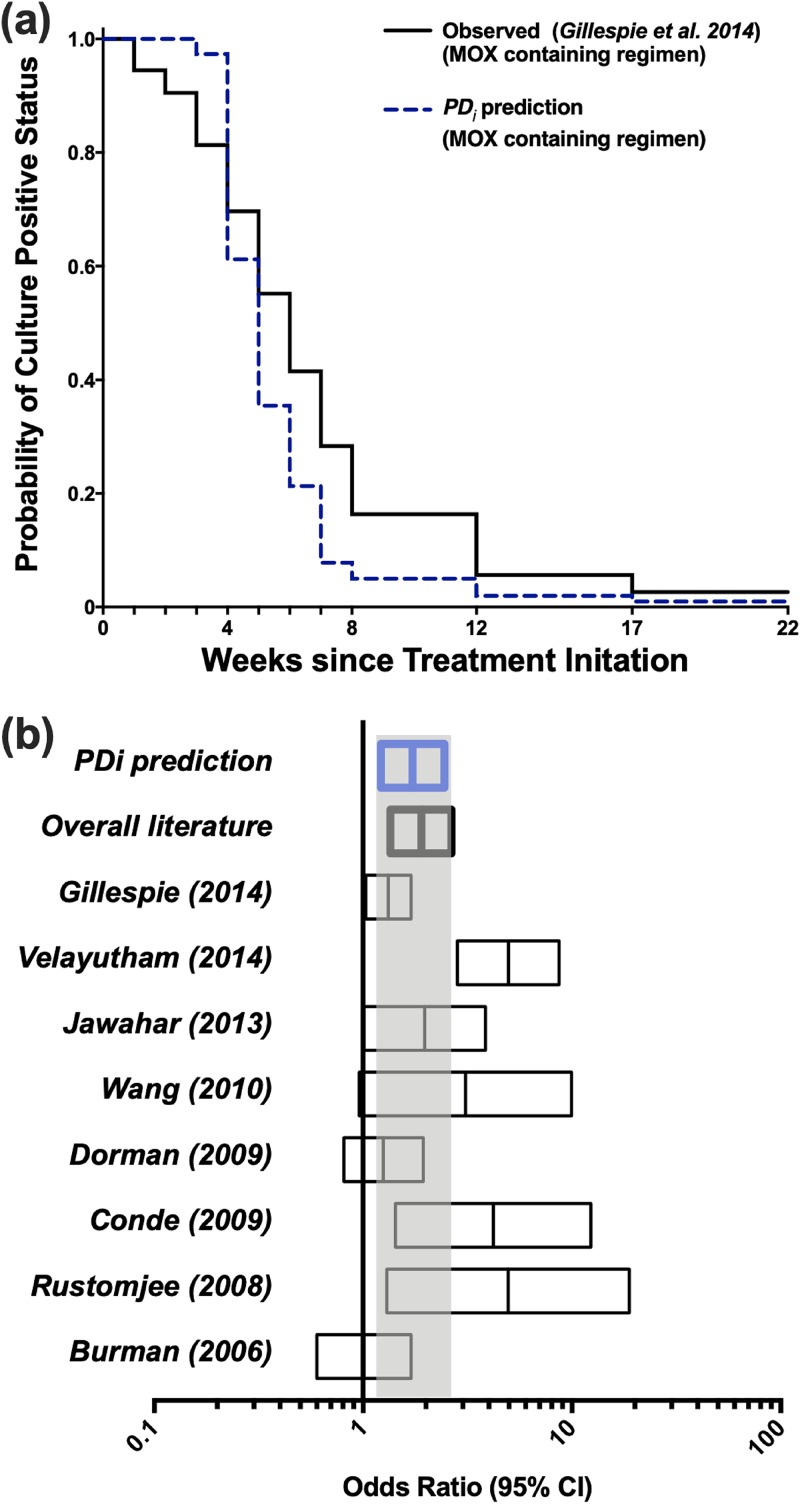
Culture conversion rates and forest plot of odds ratios. (a) Observed culture conversion rates from patients treated with MXF, RIF, EMB, and PZA for 4 months in a clinical study by Gillespie et al. (11) (solid black line) compared to our PDi prediction for the same dosing regimen over the same duration (dashed blue line). (b) Forest plot showing odds ratios from eight clinical studies (13) compared to odds ratio calculated using our PDi-based Monte Carlo simulations. The gray area represents the range of PDi prediction.

To compare our predictions with the clinical literature, we used Monte Carlo simulations to generate hypothetical treatment outcomes when using the same regimens previously used in clinical trials. PDi modeling predicted that within 8 weeks 96% of the patients would achieve culture-negative status when INH or ETB is replaced with 400 mg of MXF (the PK/PD properties of partner drugs are based on reference 22, as shown in Table S1). Our results concur with several clinical studies, as displayed in Table S2 and Fig. 4. PDi-based odds ratios were estimated to be 1.726 (confidence interval [CI] = 1.216 to 2.45), which is comparable to reported or calculated ratios from clinical literature data (Table S2, Fig. 4). PDi prediction of culture conversion rates for MXF containing regimens seems to be marginally faster than that observed in the most comprehensive study (11). However, our prediction is in line with the median of eight different clinical studies over 8 weeks (Fig. 4b and Table S2).

Table S3 displays the sensitivity analysis of the PDi modeling (see Materials and Methods), which indicates that the maximal kill rate (*E*_max_) of MXF is the most influential parameter upon bacillary clearance. The second most significant parameter is the initial intracellular burden the patient presents at the clinic, concurring with previous clinical studies (28, 29), followed by the PK parameters and MXF drug potency (EC_50_). The potency and PK parameters of RIF ranked lower than MXF parameters in our sensitivity analysis. The parameters for other partner drugs (INH, EMB, or PZA) played a negligible role in the overall outcome of the simulations.

### At higher concentrations, LVX is predicted to be as effective as MXF at killing *M. tuberculosis*.

Since the PDi model is ultimately based on pulmonary exposure (i.e., concentration in ELF), we compared the overall PK properties and the ELF exposures of MXF, CIP, and LVX (Table S4). Simulations predict MXF to have superior PK properties due to its relatively high AUC level in the plasma, and it displays the highest accumulation in the ELF. Table S4 shows that LVX at a higher dose of 750 mg/day would achieve a pulmonary PK profile very similar to that observed for MXF at 400 mg/day. Assuming linear PK for LVX, 500 mg/day is expected to achieve ELF exposure inferior to 400 mg/day of MXF. At a 1,000-mg dose, LVX has a comparable ELF AUC value (221.7 mg ⋅ h/liter) compared to a standard dose of MXF of 400 mg/day (173.1 mg ⋅ h/liter). Systematic PK parameters were chosen from previously reported data (per Table S4).

**FIG 5:**
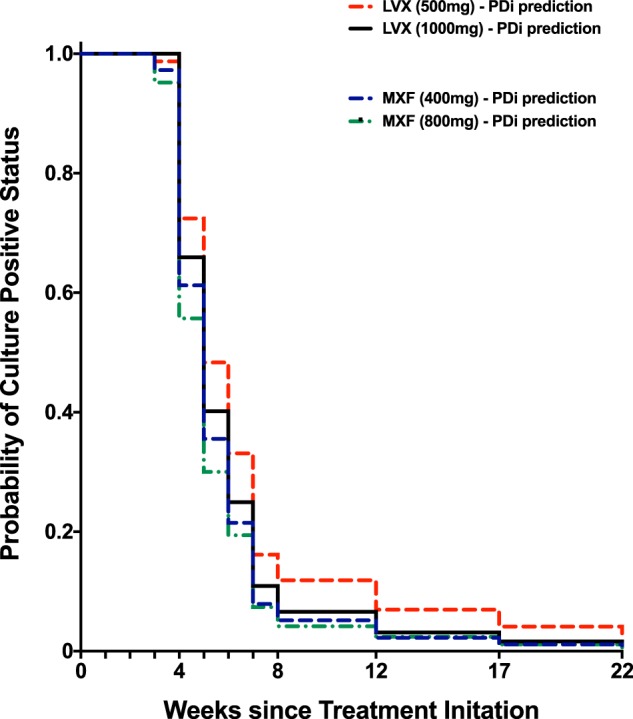
PDi predictions of culture conversion rates for MXF (400 mg daily, 4 months, dashed blue line) versus LVX (1,000 mg daily, 4 months, solid black line), LVX (500 mg daily, 4 months, dashed red line), and MXF (800 mg daily, dashed green line).

Simulating various doses of LVX and MXF and integrating this with our imaging-based PDi data reveals that an increase of LVX from 500- to 1,000-mg results in a remarkable improvement in activity (88% of patients were predicted to achieve negative culture conversion at 8 weeks with 500 mg of LVX versus 93% of patients predicted to achieve negative culture conversion at 8 weeks with 1,000 mg of LVX; Fig. 5). In contrast, we predict that an increase in the dose of MXF from 400 to 800 mg would result in a more modest improvement (95% of patients would achieve culture-negative status with 400 mg of MXF versus 96% with 800 mg of MXF) (Fig. 5).

### Using PDi-based modeling to predict TB treatment duration and risk of relapse.

**FIG 6:**
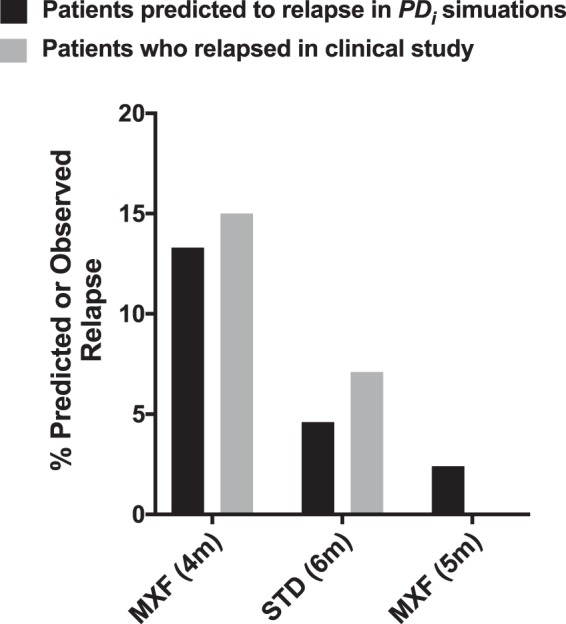
Predicted or observed relapse rates. The percentages of patients predicted to relapse (based on late culture conversion status) (black bars) compared to observed relapse rates, as observed in Gillespie et al. (11) (gray bars), are shown. The observed relapse rates are based on both MXF arms in the clinical study. 4m, 4 months; 6m, 6 months; 5m, 5 months (data for 5-month MXF are not available).

As described, fluoroquinolones have been clinically assessed in efforts to shorten standard therapy (7, 20, 30). We believe culture conversion is related to relapse rates and hypothesize that after culture conversion, further therapy is required to kill hidden/recalcitrant bacilli populations. Figure 6 displays how many patients would hypothetically be at risk of disease relapse when comparing the standard regimen to a MXF arm for 4 or 5 months. We hypothesize that this is directly related to the time taken to culture convert during treatment, which will differ between all patients. As shown, 6% of patients in the standard treatment arm will culture convert late (<80 days before end of treatment) and therefore not receive treatment for 80 days postconversion compared to 15% in the 4-month MXF and 4% in the 5-month MXF arm.

## DISCUSSION

PDi modeling has previously been utilized for the prediction of treatment outcome for TB patients receiving standard or high-dose RIF therapy (22, 31) Here, we used a similar approach to predict treatment outcomes using fluoroquinolone-based regimens. Fluoroquinolones have been considered by many to be a means to shorten the current standard therapy (7, 20, 30).

We have presented intracellular killing kinetics for six different fluoroquinolone drugs assessed using two methods. The first method is useful for rapid ranking of the potency of the various drugs resulting in concentration-effect relationships (Table S1). This allows for the identification of the best candidates for further analysis. The results demonstrated that four of the six fluoroquinolones have similar potencies (MXF, LVX, CIP, and SPX). Despite RIF, our control compound, being superior overall, MXF displayed a similar maximal kill rate in both the live fluorimetry and the fixed Operetta assay. However, patients treated with a 4-month regimen containing MXF showed higher rates of disease relapse despite achieving faster culture conversion in comparison to standard treatments (11). MXF, however, has superior PK properties compared to a standard regimen of RIF (which is currently dosed suboptimally), especially in its accumulation in the lung and ELF. The high accumulation levels compensate for the lower potency compared to RIF and leads to better overall effect in Monte Carlo simulations, predicting a median culture conversion time of 31 days compared to 56 days for standard treatment.

Our intracellular data were in agreement with data from a murine macrophage study, showing that NOX and OXF are significantly less potent than MXF (32). While SPX displayed the lowest EC_50_, cytotoxicity was observed, as reported in the literature (33); therefore, it was eliminated from further investigation. MXF, CIP, and LVX showed similar potency and maximal kill rates. CIP displayed an EC_50_ similar to that for MXF in our study, but at 500 mg it has 10-fold lower exposure in the ELF compared to MXF at 400 mg (25). Therefore, only MXF and LVX were considered further in our analyses. A 1,000-mg dose of LVX has a comparable ELF AUC value (221.7 mg ⋅ h/liter) compared to a standard dose of MXF of 400 mg/day (173.1 mg ⋅ h/liter, Table S4) and that at this higher dose, the clinical efficacy of LVX, in terms of culture conversion rates, is predicted by the PDi-based modeling to be comparable to MXF (Fig. 5). This observation is consistent with a recent clinical study comparing MXF (400 mg) to high-dose (750 mg) LVX, demonstrating that both regimens result in a similar clinical outcome (34, 35). These data support that, in the absence of any safety concerns, LVX should be further tested at higher doses.

Similar to the work described previously (22), we show that it is the intracellular kill rate of MXF that limits the reduction in CFU burden, even when the intracellular population represents just 5% of the overall population (within our model 95% represented by extracellular *M. tuberculosis*). Figure S4 displays the biexponential nature of CFU reduction which, in our simulation, is a direct result of having two separate populations (extracellular and intracellular) (Fig. S4).

That our PDi-based modeling approach (using short-term measurements of intracellular *M. tuberculosis* killing) is able to predict long-term clinical responses is perhaps not surprising when it is considered that the majority of patients (ca. 80%) culture convert within ca. 8 weeks of treatment (36). We have previously shown that clinical biphasic treatment responses can be explained by an initial reduction in extracellular *M. tuberculosis*, followed by a second, slower phase of bacillary clearance that largely corresponds to the killing dynamics of intracellular (macrophage) *M. tuberculosis* (22, 37). The duration of this second slower bacillary clearance rate is typically 5 to 6 weeks. Therefore, the PDi-based modeling approach should be viewed as a predictive tool to determine the clinical response over this shorter time frame, which is nonetheless a critical clinical feature to assess drug efficacy. Clearly, the PDi-based modeling approach does not take into account the response to treatment of slow growing/dormant bacilli, which are thought to be relevant to TB treatment outcome (38). However, given the strong agreement in the PDi-based modeling with observed clinical bacillary clearance responses, it is our view that these important PD and PK considerations are more relevant in terms of predicting microbiological treatment outcomes and potentially treatment relapse rates. As described, this study proposes that a useful proxy to estimating disease relapse is the treatment duration after culture conversion, and therefore knowledge of bacillary clearance rates for drugs and drug combinations can be used to inform treatment outcome and relapse rates.

### Should MXF be introduced to the standard treatment?

Incorporating MXF into treatment regimens results in faster clearance rates of bacilli compared to standard treatment in eight different clinical studies (13). As mentioned earlier, Gillespie et al. showed that a 4-month MXF-based treatment results in a higher relapse rate (11, 21). Superior culture conversion results indicate that relapse rates should be lower in the MXF arm. PDi simulations can accurately predict the percentage of patients reaching culture-negative status compared to observed clinical findings (Table S2). In addition, sensitivity analysis (Table S3) indicates that MXF in these regimens is the main driver of activity in combination treatments, and its effect significantly supersedes that of RIF, thus explaining the accelerated clearance of bacilli when MXF is introduced to the drug regimen.

However, the culture conversion rates in clinical findings did not predict the high relapse rate in the MXF arm that was reported in Gillespie et al. in 2014 (11). One explanation for this disparity between bacilli clearance rates and disease relapse could be related to the length of time treatment is received after achieving negative culture conversion. This will vary between individual patients. We hypothesize that each patient requires treatment exceeding 80 days following culture conversion. For example, according to our PDi predictions, in the 4-month MXF arm 12 to 17% of patients will be at risk for relapse since they will culture convert late and receive treatment for <80 days after culture converting (Fig. 6). In contrast, only 7% will be at risk of relapse in the standard 6-month arm. The remaining 93% will remain on treatment for >80 days after culture conversion. We have suggested an 80-day duration after culture conversion because this is the duration that predicts 15% relapse rate, which agrees with relapse rates observed in the Gillespie et al. clinical trial with MXF-containing regimens. This correlation between delayed culture conversion and relapse rate has previously been suggested (39), although this seems to be with limitations since many patients with delayed culture conversion might still have favorable outcomes. Based on our data and simulations, introducing MXF into the standard regimen could lead to a reduced treatment period, e.g., from 6 to 5 months. These findings further support the notion of individualized therapy, where patients with late culture conversion could receive treatment for longer durations than those with early culture conversion (40).

### Conclusions.

Our preclinical model may offer an insight into the performance of compounds with the overall aim of reducing the TB treatment period down from 6 months. By adding MXF into the regimen, we predicted the treatment outcome and could offer recommendations for its use in the clinic. Although results from previous clinical trials were disappointing when substituting MXF into the standard regimen, it is a well-tolerated compound with high antimycobacterial and favorable ELF properties. Based on our modeling, MXF and LVX have the potential to shorten the treatment period, and our data aligns with independent clinical results. The individual time frame in which a patient converts to a culture-negative status is particularly important when determining treatment duration, and a more individual-based therapy would be highly beneficial in reducing treatment duration and potentially in reducing relapse rates.

## MATERIALS AND METHODS

### Chemical compounds.

The fluoroquinolones moxifloxacin (MXF), levofloxacin (LVX), norfloxacin (NOX), ofloxacin (OFX), sparfloxacin (SPX), and ciprofloxacin (CIP) were purchased from Sigma, UK. All compounds were made up in dimethyl sulfoxide (Sigma).

### Mycobacterial strain, growth, and macrophage growth.

*M. tuberculosis* H37Rv expressing the far-red reporter mCherry was used in this study (H37Rv-mCherry) (22). Aliquots of H37Rv-mCherry was precultured aerobically at 37°C in Middlebrook 7H9 broth (Difco) supplemented with 0.05% (vol/vol) Tween 80 (Sigma), 0.2% (vol/vol) glycerol, 10% oleic acid-albumin-dextrose-catalase (OADC; 7H9), and 50 mg/liter hygromycin (Sigma) with magnetic stirrers. THP-1 cells were routinely cultured in RPMI 1640 supplemented with l-glutamine, NaHCO_3_ (Gibco), and 10% heat-inactivated fetal bovine serum (HI-FBS; Gibco) at 37°C and 5% CO_2_.

### Macrophage infection assay.

THP-1 cells were differentiated in Perkin-Elmer CellCarrier-96 plates, seeded at 5 × 10^5^ cells per well, and differentiated for 72 h in supplemented RPMI 1640 and 100 ng/ml phorbol 12-myristate 13-acetate (PMA; Sigma) at 37°C and 5% CO_2_. Differentiated THP-1 cells were infected with H37Rv-mCherry in suspension at a multiplicity of infection of 1:5 in FluoroBrite Dulbecco modified Eagle medium (DMEM) supplemented with 10% HI-FBS and l-glutamine for 24 h at 37°C. For optimal macrophage environment, each 96-well plate was covered with a Breath-EASIER sealing membrane (Sigma) to allow gaseous exchange but maintaining containment level 3 safety regulations. This has been refined from our previous work, improving the THP-1 cell growth conditions. After 24 h, the cells were washed, and the drugs at the required concentrations were added in FluoroBrite DMEM to a total volume of 200 μl. Infected cells were incubated for up to 144 h.

Extracellular-grown (planktonic) *M. tuberculosis* kill kinetics were obtained by incubating *M. tuberculosis* H37Rv in the presence of test drug, followed by plating to obtain CFU. *M. tuberculosis* was cultured at 37°C in Middlebrook 7H9 broth (Difco) supplemented with 0.05% (vol/vol) Tween 80 (Sigma), 0.2% (vol/vol) glycerol, 10% OADC (7H9), and 50 mg/liter hygromycin (Sigma) with magnetic stirrers to the mid-log-growth phase before 2-fold dilutions of test drug ranging from 15,360 to 30 ng/ml were added. A sample of culture was plated at 0 h before drug was added to obtain the initial bacterial count. After the addition of drug, 2-ml aliquots of bacterial culture with magnetic stirrers were incubated at 37°C in complete media. At defined time intervals of 24, 48, 72, 96, and 168 h, aliquots were pelleted to remove drug. These were serially diluted in PBS, plated on Middlebrook 7H11 agar, and incubated at 37°C in 5% CO_2_ for 28 days. The Miles Misra method was used to determine CFU (41).

### Fluorometer drug screening, high-content image acquisition, and data analysis.

Data were generated from multiple independent experiments (*n* ≥ 3), all performed at least in triplicate, and data were produced via two methods. First, each plate was screened every 24 h for fluorescence using a Varioskan (LUX multimode reader; Thermo Scientific) at an excitation of 578 nm and emission of 610 nm, thus producing “live” fluorometer readouts. In addition, plates were fixed with 5% paraformaldehyde (Sigma) for 2 h for imaging using an Operetta (Perkin-Elmer) with a 60× High NA objective, as described previously (22).

The *Z*′-factor for the Varioskan assay, calculated using an equation reported by Zhang et al. (42), was 0.57 for the data set.

Unlike the Operetta-based high content imaging screen, maximum kill rates cannot be calculated from the fluorometer. Upon bacillus death, the measured linear response of *M. tuberculosis* to a compound eventually plateaus, limiting the dynamic range of the assay.

### Data analysis and modeling.

Fluoroquinolone activity was ranked using the Varioskan fluorimeter readout. An algorithm was used for drug combinations where the overall kill rate at any given time is equal to the kill rate of the drug with the highest kill rate at that given time point (this kill rate is dependent on the changing of a drug’s concentration and its constant PD parameters [EC_50_ and *E*_max_]). Hence, the model assumes that there are no positive or negative PD interactions between the drugs (31). Bacterial growth and death rates at different drug concentrations were calculated using Pmetrics GraphPad Prism as follows:

(1) M.tuberculosis count = initial M. tuberculosis count×[1−exp(−K×x)];

where *K* represents the growth rate per hour and would be a negative value if the bacterial count is decreasing over time. Each drug concentration generated an independent *K* value. This value was normalized to bacterial growth per experiment to avoid interexperimental bias, and thus the growth rate was divided by the *K* value to normalize the kill rate. Kill rates at different concentrations are then fitted to a three-parameter pharmacological model according to the following equation using GraphPad Prism:

(2) $$E=\frac{E_{\text{max}}\times C}{{\text{EC}}_{50}+C}+E_{\text{min}};$$

where *E* is the kill rate at any given concentration, *E*_max_ is the maximal kill rate of each drug, *C* is the drug concentration in mg/liter, and EC_50_ is the concentration required to achieve a half-maximal kill rate.

Extracellular-grown *M. tuberculosis* kill rates and EC_50_ values for RIF, INH, and EMB were derived from our previous study (22), whereas MXF extracellular EC_50_ (356 ng/ml) and kill rate (0.23h^−1^) values were derived in this study (Fig. S5). The LVX extracellular *E*_max_ and EC_50_ values were assumed to be similar to those for MXF for the purposes of this work.

### Modeling parameters.

Parameters for RIF, INH, PZA, and EMB were derived from our previous work (22), whereas parameters for fluoroquinolone drugs were derived from experiments described here. Table S5 summarizes the parameters used for the simulation with the corresponding references.

We observed a very strong correlation between the growth rate and the corresponding kill rate in all experiments for all drugs screened. RIF and MXF exhibited similar maximal kill rates that always varied between 1.6- and 2-fold higher than the growth rate, regardless of whether the latter was fast or slow. The consistency in the ratio between the growth and kill rates allows the correction of all data to a fixed growth rate, in all simulations, to reduce output noise while not compromising the final outcomes. Reported intracellular *M. tuberculosis* growth rates (also known as doubling times [DT]), including those reported by us, range from a DT of 21 h to a DT of 48 h (22, 43, 44), while *in vivo* rodent TB models report an intracellular DT for *M. tuberculosis* close to 25 h (45). The reason for the reported variation is not fully understood but could be partially explained by macrophage modulation of M0/M1/M2 polarization (37). For the described PDi-based modeling approach, we chose a DT of 21 h for consistency with previous studies and to avoid bias in cross-study comparisons in the future.

### Monte Carlo simulations.

Monte Carlo simulations for PK/PD predictions were performed using Pmetrics with PK parameters derived from the literature. The PK values for MXF were chosen from studies where MXF was administered with RIF due to the known PK interaction between the two drugs (23, 24). The PK values for other standard drugs were used as previously described (22).

The models utilized for Monte Carlo simulations here were as described in detail elsewhere (22, 31). Briefly, the CFU reduction in each simulated patient was driven by exposure to the drug and kill rates of each drug according to an *E*_max_ model (equation 2). Extracellular and intracellular bacilli respond differently to drug treatment, and both reservoirs were simultaneously simulated for each patient, resulting in a biexponential decrease in total CFU burden over time (Fig. S3). Overall, the drug kill rate (of all drugs in combination) is equal to the rate of the fastest-acting drug at the same time. This is determined by the epithelial lining fluid (ELF) drug exposure and its intrinsic rate of kill (as defined *in vitro*) at any given time point for each drug.

The initial clinical CFU count in each of the 1,000 simulated patient lungs was assumed to be 10^7^ CFU/ml (22). All PK profiles for analysis were assumed to follow a one-compartment model. The pulmonary levels of drug were assumed to be the driver for activity (where *C* in equation 2 represents the drug concentration in the ELF); ELF levels were estimated by using the ELF/serum ratio reported in the literature, as previously described for RIF, ETB, INH, and PZA, and an ELF ratio of 5.2 was estimated for MXF (46). The CFU change was recorded over time at fixed intervals of 1 week over a simulated run time of 4 months (for regimens including MXF, RIF, INH, and PZA) to 6 months (the standard regimen). It was assumed that a ≤10 CFU/ml outcome at any given time would indicate culture conversion to be negative, as previously described (22). The number of patients converting to culture-negative status in the simulation every week was then recorded for further survival analysis. Table S5 summarizes the parameters used for the simulation with the corresponding references.

Odds ratios were calculated using IBM SPSS Statistics (version 24; property of IBM Corp.). Data generated form Monte Carlo simulation from different scenarios were compared head to head, and the odds ratio with 95% CI was estimated accordingly.

Sensitivity analysis was performed to assess the most influential parameters upon the bacillary clearance within the simulation (Table S3). The analyses were performed using the FME package (a Flexible Modeling Environment for Inverse Modeling, Sensitivity, Identifiability and Monte Carlo Analysis) with R version 3.4.2 (47). Influence of each parameter was expressed in terms of L1-norm and L2-norm measures, which rank parameters by their effect upon the simulation. The higher the L1-norm or L2-norm for a given parameter, the higher the sensitivity (48). Sensitivity analysis (Table S3) shows that MXF would be the main driver of activity in a combination of drugs. This is further corroborated by observing the changing kill rate over time for each of the partner drugs, which shows that MXF dominates as the fastest killer throughout the treatment duration, followed by RIF, whereas INH and PZA play a negligible role in the overall clearance of bacilli (Fig. S5).

### Methodological assumptions and limitations.

While fluorometry-based measurements of intracellular *M. tuberculosis* can effectively measure bacillary growth, this approach has a limited dynamic range when assessing bacillary sterilization over time when measurements fall below the minimum limit of fluorescence detection. To overcome this issue, high-content (Operetta) imaging was used to more accurately measure time-dependent bacillary sterilization for specific drugs. With regard to intracellular *M. tuberculosis* growth, during the course of this and our other studies, as well as in studies by other laboratories, we noted a variation in intracellular *M. tuberculosis* DT, as mention above under “Modeling Parameters.” To normalize for this in our mathematical modeling, we selected a DT of 21 h throughout. Furthermore, for the purpose of the mathematical modeling/simulations to predict clinical outcome, in the absence of evidence to the contrary, we also made a number of assumptions. First, it was assumed that there is no pharmacological interaction (synergy/additivity/antagonism) between the modeled drugs; therefore, the clinically observed *M. tuberculosis* sterilization rate was equivalent to the sterilization rate of the drug with the fastest sterilization rate. Second, it was also hypothesized that PD interactions of drugs against intracellular (macrophage) *M. tuberculosis* measured in *in vitro* culture were similar to those in the ELF.

It should be noted that while time-dependent intracellular *M. tuberculosis* sterilization rates and PDi PK modeling have been used in this study to predict the clinical activity of fluoroquinolones against TB, this approach generates dynamic parameters (e.g., EC_50_ and *E*_max_) that are not suitable for comparison with traditional static microbiological parameters such as the MIC.

## Supplementary Material

Supplemental file 1
