# Supplementary material for: Intracellular Pharmacodynamic Modeling Is Predictive of the Clinical Activity of Fluoroquinolones against Tuberculosis
Source: Antimicrob Agents Chemother. 2019 Dec 20;64(1):e00989-19. doi: 10.1128/AAC.00989-19 (PMC7187570; doi:10.1128/AAC.00989-19)
Supplement: Supplemental file 1 [file AAC.00989-19-s0001.pdf]

**Supplementary Figures**

## Supplementary Figure. 1

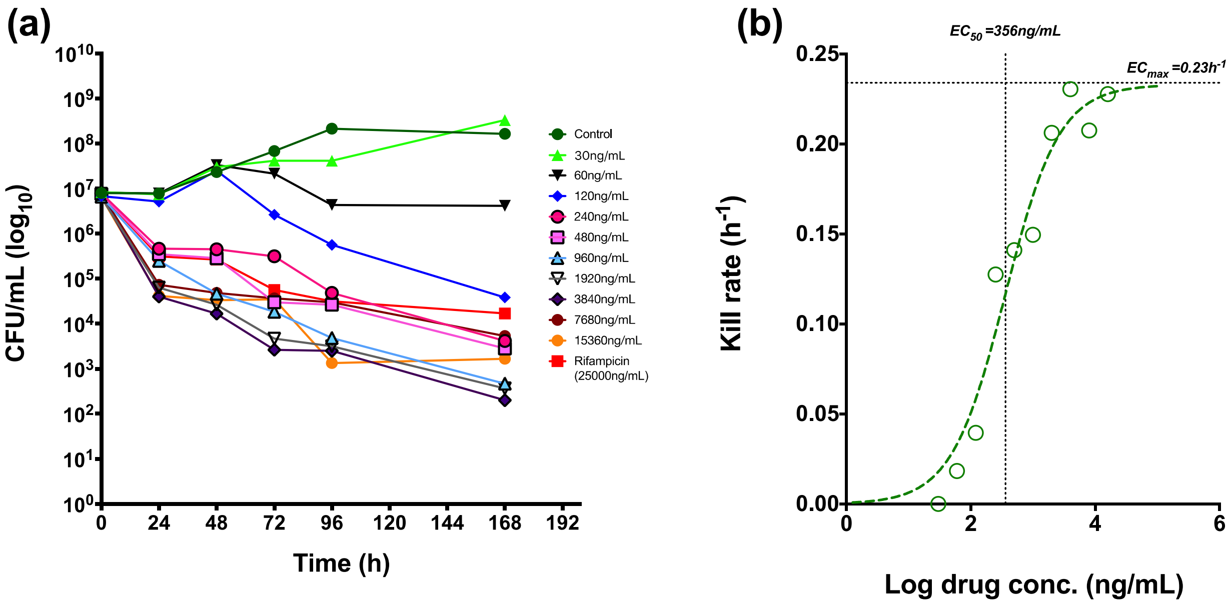


*S. Fig. 1 (a) Time-kill of extracellular (planktonic)-grown Mtb for MXF over a concentration range of 30 ng/mL-15360 ng/mL during a 168 h time period. (b) Conc.-Effect Relationship of MXF against extracellularly-grown (planktonic) Mtb.*

**Supplementary Figure. 2**

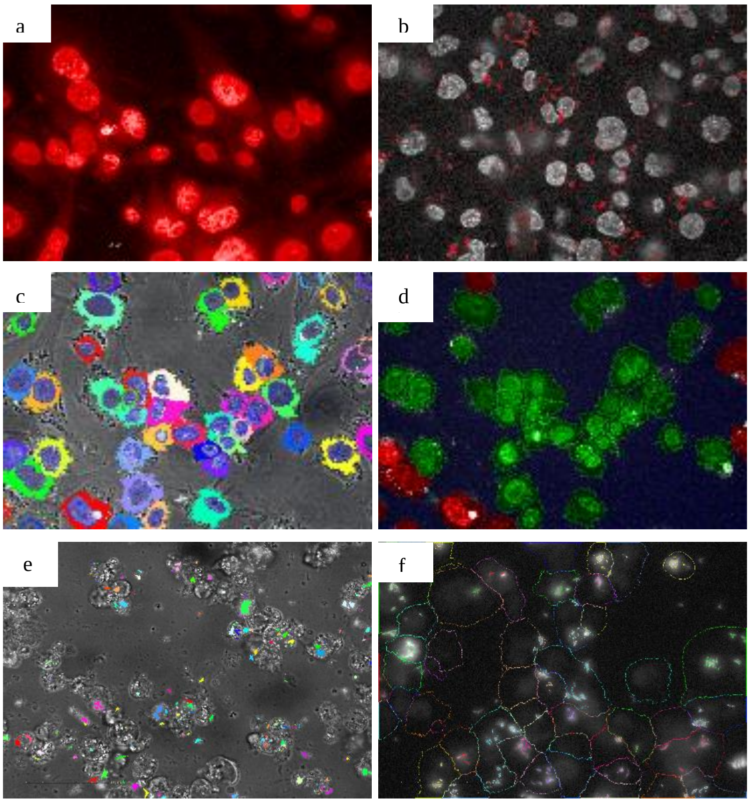


*S. Fig. 2 An example of the Harmony Software analysis for the Operetta (PerkinElmer) (a) Detecting nuclei (b) Detecting bacilli (c) Outlining the cytoplasm (d) Disregarding non-whole cells, and how only the intracellular bacilli are detected (e-f). All scale bars 50 µm.*

**Supplementary Figure. 3**

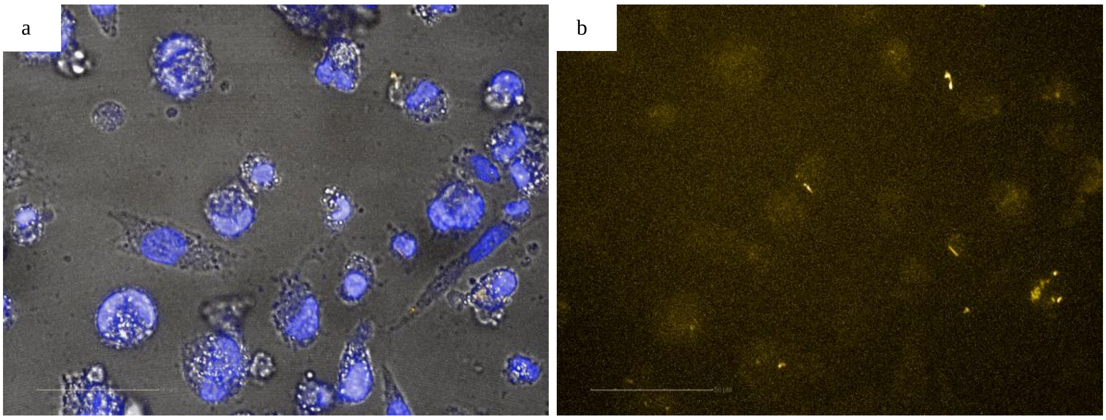


*S. Fig. 3 Fluorescent images as acquired from the Operetta (PerkinElmer) Fixed macrophages infected with Mtb H37Rv expressing the far-red reporter mCherry after 144 h with 100 mg/L MXF (a) showing full z-stack image (b) with only the bacilli displayed (as detected by the Harmony Software). All scale bars 50 µm.*

**Supplementary Figure. 4**

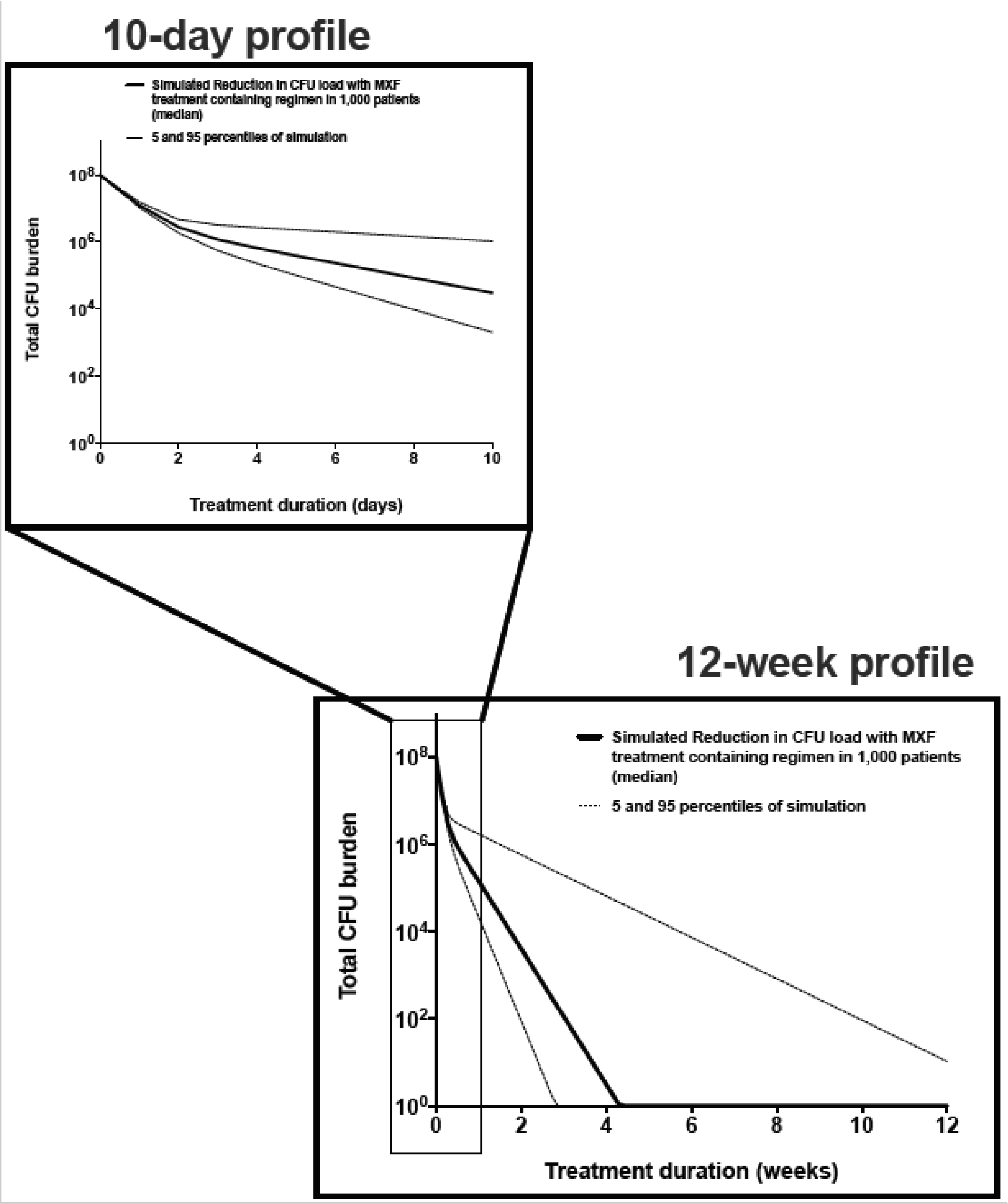


*S. Fig. 4 Monte Carlo simulation: Reduction of total CFU burden over time in a simulated population of 1000 people. The solid line is the median of the simulation showing that 50% of patients taking MXF containing regimens will achieve culture conversion in 4-5 weeks and the dashed lines represent the 5-95 percentile of the simulation. The biexponential nature of CFU reduction is due to the differing kill rates against intracellular and extracellular bacteria (the top box shows the initial 10 days of treatment displaying the fastest rate of reduction).*

**Supplementary Figure. 5**

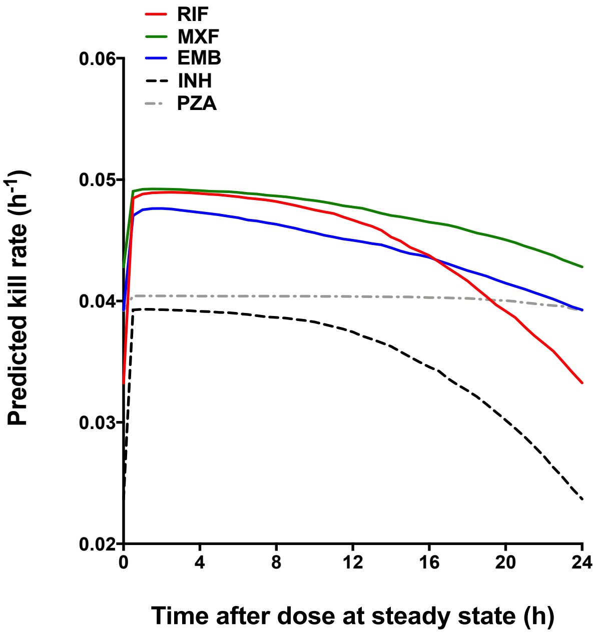


*S. Fig. 5. The changing kill rate of each drug over time, after dose at steady state. Kill rates change as drug levels reduce as drug it is cleared from the body after dosing.*

**Supplementary Table 1.**

**Summary of selected fluoroquinolone intracellular pharmacodynamic properties.**

*Results show median values for fits with 95 confidence intervals in brackets. n=12-18 from ≥ 3 independent biological experiments for each compound.*

| ***Drug*** | | ***$EC_{50}$ (mg/L)*** |
|---|---|---|
| Moxifloxacin | MXF | 0.238 (124-455) |
| Ciprofloxacin | CIP | 0.259 (162–415) |
| Sparfloxacin | SPX | 0.051 (N/A) |
| Levofloxacin | LVX | 0.382(91-1541) |
| Ofloxacin | OFX | 1.41 (179-1112) |
| Norofloxacin | NOX | 1.71 (233-9456) |

**Supplementary Table. 2.**

*Comparison between PDi modelling results and different clinical outcomes defined as percentage of patients who achieve negative culture conversion in 8 weeks in comparison to standard treatment.*

| ***Study*** | ***% Culture Conversion @ 8 weeks*** | ***PDi prediction*** |
|---|---|---|
| *Burnam et al. 2006* (1) | 71% | 90% |
| *Rustomjee et al. 2008* (2) | 97% | 96% |
| *Conde et al. 2009* (3) | 95% | 96% |
| *Dorman et al. 2009* (4) | 91% | 85% |
| *Wang et al. 2010* (5) | ~94% | 96% |
| *Gillespie et al. 2014* (6) | 75% | 96% |

**Supplementary Table. 3.**

*Sensitivity analysis results showing the ranking of different parameters upon the intracellular bacillary load at the end of treatment.*

| ***Parameter*** | ***L1-norm*** | ***L2-norm*** |
|---|---:|---:|
| *$E_{max}$ (MXF)* | 757428.4 | 320895.913 |
| *Initial intracellular bacillary load* | 322909.2 | 152872.1 |
| *$E_{max}$ (RIF)* | 265586.7 | 117598.3 |
| *$K_e$ (MXF)* | 35022.7 | 14809.3 |
| *V/F (MXF)* | 27330.0 | 11646.1 |
| *$EC_{50}$ (MXF)* | 27324.2 | 11643.2 |
| *$EC_{50}$ (RIF)* | 3683.1 | 1647.0 |
| *V/F (RIF)* | 3682.5 | 1646.9 |
| *$K_e$ (RIF)* | 2016.7 | 911.1 |
| *$K_a$ (MXF)* | 325.7 | 144.9 |
| *$K_a$ (RIF)* | 230.3 | 99.9 |
| *Initial extracellular bacillary burden* | 2.7 | 0.98 |
| *POPMAX* | 0.0039 | 0.0021 |

**Supplementary Table. 4.**

*Summary of pharmacokinetic parameters of MXF, CIP and LVX as reported in different literature sources. AUC = Area under the curve from 0-inf with one dose. ELF stands for concentrations at the epithelial lining fluid.*

| ***Drug*** | ***Reported Plasma $AUC_{0-24}$ (mg.h/L)*** | ***Estimated ELF:Plasma AUC ratio (Penetration ratio)*** | ***References*** |
|---|---|---|---|
| *MXF (400 mg)* | 33.3 | 5.2 | (7) |
| *CIP (500-750 mg)* | 9.8, 12.2 | 0.82 | (8) |
| *LVX (750 mg)* | 125 | 1.43 | (9) |

## Supplementary Table 5

*Parameters used in PDi modelling of treatment regimens simulated in this work. PK parameters for RIF, INH and PZA taken from* (10), *MXF taken from* (11) *LVX taken from* (12) . *ELF:Plasma ratio for RIF, EMB, INH and PZA, MXF and LVX taken from data reviewed in* (13). *$E_{max}$ and $EC_{50}$ parameters for RIF, INH, EMB and PZA were from* (14). *Whereas $E_{max}$ and $EC_{50}$ values for MXF and LVX were derived from in vitro studies described in this work.*

| Treatment | Parameter | Value* |
|---|---|---|
| **Control** | **$Kg_{max}$ ($h^{-1}$ (D.Time)** | 0.033 (21.0 h) |
| **Rifampicin** | **$E_{max}$ ($h^{-1}$)** | 0.055 |
| | **$EC_{50}$ (ng/mL)** | 18.4 |
| | **V/F (L/kg)** | 1.0 |
| | **CL/F (L/h/kg)** | 0.41 |
| | **ELF:Plasma ratio** | 0.26 |
| **Ethambutol** | **$E_{max}$ ($h^{-1}$)** | 0.053 |
| | **$EC_{50}$ (ng/mL)** | 79.5 |
| | **V/F (L/kg)** | 10.3 |
| | **CL/F (L/h/kg)** | 0.78 |
| | **ELF:Plasma ratio** | 1.03 |
| **Isoniazid** | **$E_{max}$ ($h^{-1}$)** | 0.041 |
| | **$EC_{50}$ (ng/mL)** | 32.1 |
| | **V/F (L/kg)** | 2.1 |
| | **CL/F (L/h/kg)** | 0.45 |
| | **ELF:Plasma ratio** | 3.53 |
| **Pyrazinamide** | **$E_{max}$ ($h^{-1}$)** | 0.043 |
| | **$EC_{50}$ (ng/mL)** | 45.5 |
| | **V/F (L/kg)** | 0.71 |
| | **CL/F (L/h/kg)** | 0.070 |
| | **ELF:Plasma ratio** | 19.2 |
| **Moxifloxacin** | **$E_{max}$ ($h^{-1}$)** | 0.055 |
| | **$EC_{50}$ (ng/mL)** | 238.0 |
| | **V/F (L/kg)** | 2.05 |
| | **CL/F (L/h/kg)** | 0.20 |
| | **ELF:Plasma ratio** | 5.2 |
| **Levofloxacin** | **$E_{max}$ ($h^{-1}$)** | 0.055 |
| | **$EC_{50}$ (ng/mL)** | 382 |
| | **V/F (L/kg)** | 1.28 |
| | **CL/F (L/h/kg)** | 0.12 |
| | **ELF:Plasma ratio** | 1.43 |

**Supplementary References**


1. Burman WJ, Goldberg S, Johnson JL, Muzanye G, Engle M, Mosher AW, Choudhri S, Daley CL, Munsiff SS, Zhao Z, Vernon A, Chaisson RE. 2006. Moxifloxacin versus ethambutol in the first 2 months of treatment for pulmonary tuberculosis. Am J Respir Crit Care Med 174:331–338.
2. Rustomjee R, Lienhardt C, Kanyok T, Davies GR, Levin J, Mthiyane T, Reddy C, Sturm AW, Sirgel FA, Allen J, Coleman DJ, Fourie B, Mitchison DA, Gatifloxacin for TB study team. 2008. A Phase II study of the sterilising activities of ofloxacin, gatifloxacin and moxifloxacin in pulmonary tuberculosis. Int J Tuberc Lung Dis 12:128–138.
3. Conde MB, Efron A, Loredo C, De Souza GR, Graca NP, Cezar MC, Ram M, Chaudhary MA, Bishai WR, Kritski AL, Chaisson RE. 2009. Moxifloxacin versus ethambutol in the initial treatment of tuberculosis: a double-blind, randomised, controlled phase II trial. Lancet 373:1183–1189.
4. Dorman SE, Johnson JL, Goldberg S, Muzanye G, Padayatchi N, Bozeman L, Heilig CM, Bernardo J, Choudhri S, Grosset JH, Guy E, Guyadeen P, Leus MC, Maltas G, Menzies D, Nuermberger EL, Villarino M, Vernon A, Chaisson RE, Tuberculosis Trials C. 2009. Substitution of moxifloxacin for isoniazid during intensive phase treatment of pulmonary tuberculosis. Am J Respir Crit Care Med 180:273–280.
5. Wang JY, Wang JT, Tsai TH, Hsu CL, Yu CJ, Hsueh PR, Lee LN, Yang PC. 2010. Adding moxifloxacin is associated with a shorter time to culture conversion in pulmonary tuberculosis. Int J Tuberc Lung Dis 14:65–71.
6. Gillespie SH, Crook AM, McHugh TD, Mendel CM, Meredith SK, Murray SR, Pappas F, Phillips PPJ, Nunn AJ. 2014. Four-Month Moxifloxacin-Based Regimens for Drug-Sensitive Tuberculosis. N Engl J Med 371:1577–1587.
7. Soman A, Honeybourne D, Andrews J, Jevons G, Wise R. 1999. Concentrations of moxifloxacin in serum and pulmonary compartments following a single 400 mg oral dose in patients undergoing fibre-optic bronchoscopy. J Antimicrob Chemother 44:835–838.
8. Wise R, Baldwin DR, Andrews JM, Honeybourne D. 1991. Comparative pharmacokinetic disposition of fluoroquinolones in the lung. J Antimicrob Chemother 28:65–71.
9. Rodvold KA, Drusano GL, Preston SL, Gotfried MH, Danziger LH, Rodvold KA. 2002. Levofloxacin Penetration into Epithelial Lining Fluid as Determined by Population Pharmacokinetic Modeling and Monte Carlo Simulation. Antimicrob Agents Chemother 46:1–5.
10. Boeree MJ, Heinrich N, Aarnoutse R, Diacon AH, Dawson R, Rehal S, Kibiki GS, Churchyard G, Sanne I, Ntinginya NE, Minja LT, Hunt RD, Charalambous S, Hanekom M, Semvua HH, Mpagama SG, Manyama C, Mtafya B, Reither K, Wallis RS, Venter A, Narunsky K, Mekota A, Henne S, Colbers A, van Balen GP, Gillespie SH, Phillips PPJ, Hoelscher M. 2017. High-dose rifampicin, moxifloxacin, and SQ109 for treating tuberculosis: a multi-arm, multi-stage randomised controlled trial. Lancet Infect Dis 17:39–49.
11. Nijland HMJ, Ruslami R, Suroto AJ, Burger DM, Alisjahbana B, van Crevel R, Aarnoutse RE. 2007. Rifampicin Reduces Plasma Concentrations of Moxifloxacin in Patients with Tuberculosis. Clin Infect Dis 45:1001–1007.
12. Peloquin CA, Hadad DJ, Molino LP, Palaci M, Boom WH, Dietze R, Johnson

JL. 2008. Population pharmacokinetics of levofloxacin, gatifloxacin, and moxifloxacin in adults with pulmonary tuberculosis. Antimicrob Agents Chemother 52:852–857.

13. Rodvold KA, George JM, Yoo L. 2011. Penetration of anti-infective agents into pulmonary epithelial lining fluid: focus on antibacterial agents. Clin Pharmacokinet 50:637–664.

14. Aljayyoussi G, Jenkins VA, Sharma R, Ardrey A, Donnellan S, Ward SA, Biagini GA. 2017. Pharmacokinetic-Pharmacodynamic modelling of intracellular Mycobacterium tuberculosis growth and kill rates is predictive of clinical treatment duration. Sci Rep 7:502.
